# Coherent control of symmetry breaking in transverse-field Ising chains using few-cycle pulses

**DOI:** 10.1515/nanoph-2022-0811

**Published:** 2023-03-01

**Authors:** Nikolai D. Klimkin, Alexey N. Rubtsov, Misha Ivanov

**Affiliations:** Max-Born-Institute, Max-Born Straße 2A, D-12489 Berlin, Germany; Russian Quantum Center, Bolshoy Bulvar 30, bld. 1, Moscow 121205, Russia; Faculty of Physics, Lomonosov Moscow State University, Leninskie Gory 1-2, Moscow 119991, Russia; Department of Physics, Humboldt University, Newtonstraße 15, D-12489 Berlin, Germany; Blackett Laboratory, Imperial College London, South Kensington Campus, SW7 2AZ London, UK

**Keywords:** correlated systems, spontaneous symmetry breaking, strong-field physics

## Abstract

Coherent control of quantum systems with phase-stable pulses offers enticing new opportunities for lightwave electronics. Here we extend this approach to many-body systems with spin degrees of freedom, demonstrating that a single few-cycle control pulse can create a sizable population asymmetry between the two degenerate polar ground states of the Ising model. This opens a route for femtosecond-scale data processing and storage, allowing one to control the final ground state of a correlated system in an all-optical way.

## 1 Introduction

**Figure 1:**
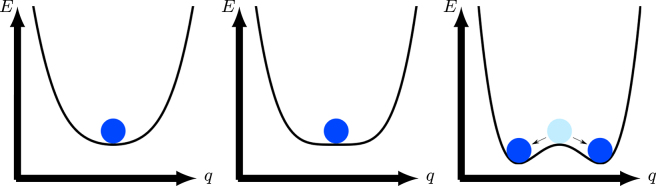
Pictorial representation of the principle of spontaneous symmetry breaking with *q* and *E* representing generalized coordinate and energy. Although the potential and therefore the equations of motion preserve space inversion symmetry, the low-energy trajectories of the particle defined by them may still break it.

Correlated many-body systems offer many examples of spontaneous symmetry breaking, see Figure 1. For example, the interaction between adjacent spins in an Ising chain aligns them either up or down, giving rise to two energetically degenerate ferromagnetic ground states of opposite polarity. Spontaneous symmetry breaking corresponds to population of one of these two (equally probable) states. Once populated, a given state does not mix with its counterpart. In thermal equilibrium, however, both states are equally populated.

Here we show how a short pulse with no DC component can selectively depopulate one of these two states, achieving unidirectional conversion between them, with asymmetric many-body excitations generated on the sub-cycle time-scale via highly nonlinear interaction with a tailored pump pulse. The induced asymmetry is controlled by the sub-cycle structure of the pulse and is preserved during and after thermalization, leading to preferential population of the ground state with the desired polarity, long after the end of the pulse. Thus, our scheme takes advantage of both the sub-cycle control over the asymmetry of the individual oscillations of the driving pulse and the symmetrizing many-body interactions after the pulse. From the fundamental perspective, our work extends the ideas of coherent control to strongly correlated systems. From the applied perspective, our work suggests a novel route to ultrafast manipulation of robust memory cells.

At a first glance, our result may appear counter-intuitive. Indeed, a pulse incident on a medium excites a current whose symmetry is defined by the symmetry properties of both the field and the medium. If the neighboring half-cycles of the incident pulse are perfect inverted images of each other, and the medium itself is inversion-symmetric, the generated excitation should exhibit no asymmetry. The medium thus acts as a perfectly balanced interferometer, where different half-cycles correspond to different arms, and the excitations generated in the successive half-cycles of the pulse interfere destructively for all even-order harmonics of the excited current. One needs to break either the symmetry between neighboring half-cycles or the inversion symmetry of the system to make the “arms” unbalanced. This would allow for the observation of phenomena prohibited in the case of perfect inversion symmetry, such as even harmonic generation and optical rectification.

The most intuitive way of breaking the dynamic half-cycle inversion symmetry is by using phase-locked mixed-color fields with differing parity, typically *ω* and 2*ω*. The phase between the two fields then becomes the control knob of the induced response, steering the formation of parity-breaking currents in atoms [1], quantum wells [2], and bulk semiconductors [3, 4]. The same principle can be transferred to the ultrafast domain by using few-cycle fields with nearly-octave spectral bandwidth.

Indeed, in nearly single-cycle driving pulses, the neighboring half-cycles of the pulse can be unbalanced even if the symmetry is present in the pulse on average, i.e. the field contains no DC component. The imbalance manifests in the nonlinear interaction and is controlled by the phase of the carrier oscillations under the envelope (the carrier envelope phase, CEP). Changes in the CEP control the balance between sub-cycle excitation processes occurring during neighboring half-cycles, inducing asymmetric response. In the frequency domain, this control can be understood as the interference between multiphoton transitions with different parity; such interference inevitably arises in few-cycle pulses with the spectral bandwidth comparable to an octave. In systems with inversion symmetry, CEP-stable pulses have been demonstrated to give rise to asymmetric photoelectron ionization [5–8], control electron localization in dissociating D_2_ molecules [9, 10], and excite CEP-dependent photocurrents in monolayer graphene [11]. In inversion-asymmetric systems, exemplified by polar molecules, orientation-selective ionization has been demonstrated with CEP-controlled ultrashort pulses [12, 13]. CEP-stable pulses have also been demonstrated to create valley-selective excitations in hBN [14].

The above discussion applies to all systems generating an electronic response. However, we expect correlated systems to be more sensitive to the field’s symmetry due to the positive feedback caused by electronic correlations. Indeed, as demonstrated by recent works [15–17], it is the field-driven movement of collective excitations that becomes the main underlying feature in many-body correlated systems. These excitations, when driven into a high-energy metastable state, are subject to spontaneous symmetry breaking, amplifying any asymmetry introduced into the system [18, 19]. The importance of coupling between several degrees of freedom in enhancing symmetry breaking has also been found in molecules We thus extend the idea of symmetry collapse in two-level systems [20] to the correlated systems, using the doubly degenerate ground state of the Ising model as an example.

We note that the Ising spin chain is a generic model, which can represent not only magnetic spins, but also electric dipoles, coupled two-level systems, other pseudospins, etc. The driving field can therefore be either a magnetic pulse or an optical electric field pulse. In the context of nonlinear interaction directly with magnetic spins, we would like to bring the reader’s attention to the recently demonstrated feasibility of strong magnetic pulses generated on-demand by spatially-modulated light [21, 22].

## 2 Methods

The Ising model consists of multiple spins localized on sites arranged in a ring, connected by a spin-spin interaction. In the results presented below, the number of these spins *N* is set to 12. However, we have verified numerically that the quantitative difference between this case and the one with *N* = 16 is negligible. The model is described by the basic transverse Ising Hamiltonian (below), where <ij> denotes summation over neighboring sites *i* and *j*, *σ* ^*α*^ denotes the respective Pauli matrix for *α* ∈ {*x*, *y*, *z*}:

(1) $${\hat{H}}_{0}=J\underset{<ij>}{\sum }\sigma_{i}^{z}\sigma_{j}^{z}+g\underset{i}{\sum }\sigma_{i}^{x}$$

The *J* term aligns the spins, creating correlations between them. It gives rise to two degenerate ground states with either a ferromagnetic or an antiferromagnetic order, depending on the sign of *J*. We consider negative *J*, leading to the ferromagnetic order. The two ferromagnetic ground states are separated from excited states by a gap increasing with |*J*|. Conversely, the single-site term *g* suppresses this order, “tipping” the spins to the side. Thanks to this term, however, the “all-down” ground state of the system |↓↓ … ↓⟩ mixes with less oriented states like |↑↓ … ↓⟩. This decreases the gap according to Δ = 2|(|*J*| − |*g*|)|.

When a field, characterized by an on-site interaction term *σ* ^*z*^ is applied, this creates nontrivial phase between the components of the ground state, mixing it with excited states. The same happens with the inverted, “all-up” state.

In the absence of a field, the Hamiltonian (1) can be solved exactly [23] using the Jordan–Wigner transform [24]. In this work, we set *g* = 1, with all other relevant parameters expressed in units of *g*. In particular, we set *J* = −2.4, in which case the ground state of the system is doubly degenerate and exhibits ferromagnetic order with positive nearest-neighbor correlations. We denote the two ground states |*g* ↑⟩ and |*g* ↓⟩.

**Figure 2:**
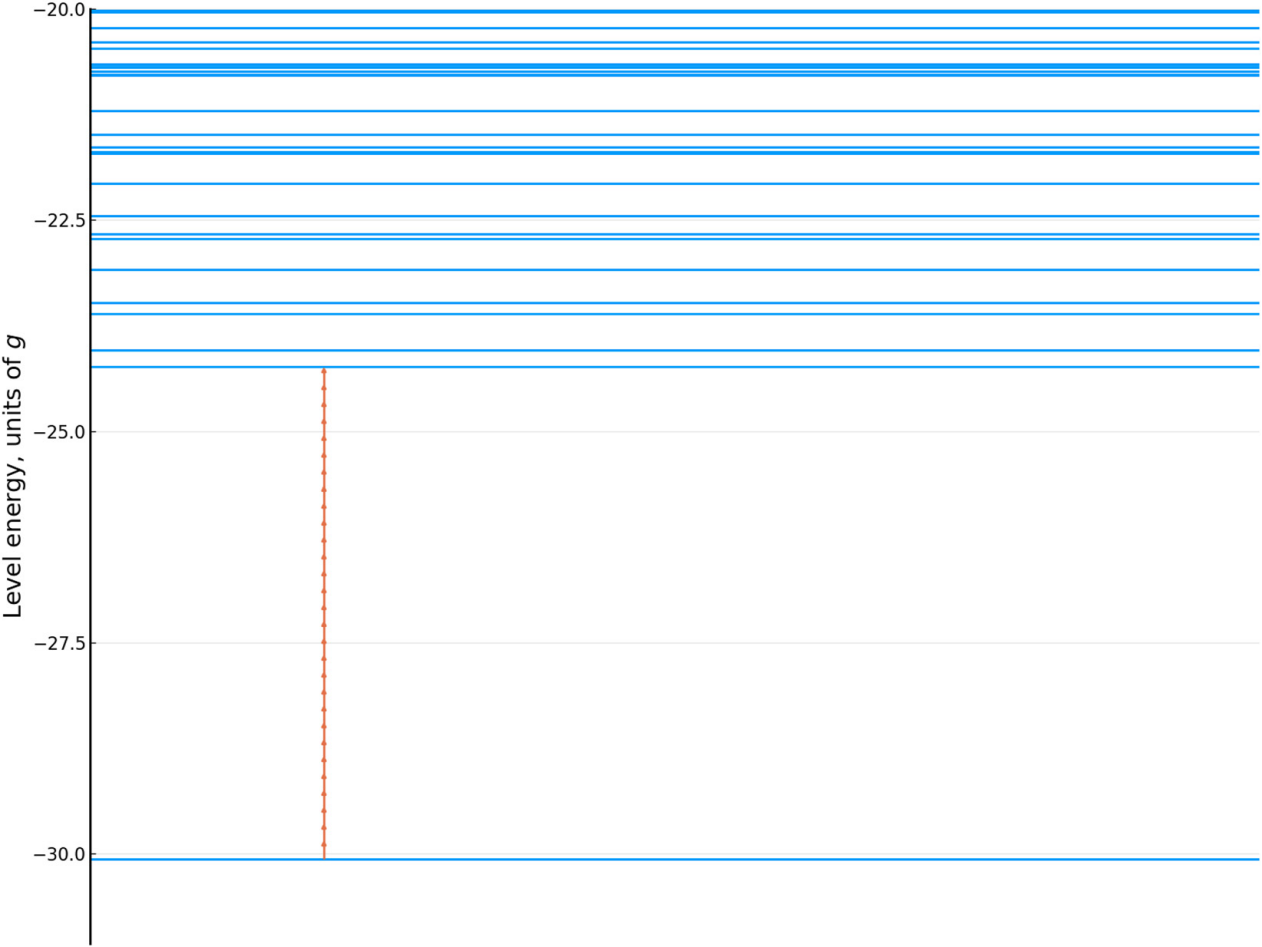
Low-energy level structure of the field-free system. A single driver field photon is marked for comparison as a red arrow.

For these parameters, the spectrum of the field-free system in units of *g* is shown in Figure 2.

We use pulses with low carrier frequency *ω*_0_ ≪ *g*, ‖*J*‖

(2) $$F\left(t\right)=\frac{\partial A}{\partial t}$$

(3) $$\begin{array}{ll} A\left(t\right) & =A_{0}f\left(t\right)\cdot \sin\left(\omega_{0}t+\varphi \right)\\ & =\frac{F_{0}}{\omega_{0}}\exp\left(-t^{2}/2\sigma^{2}\right)\cdot \sin\left(\omega_{0}t+\varphi \right) \end{array}$$

where *f*(*t*) is the pulse envelope, which we set to be Gaussian with a width characterized by *σ*. The strength of the field is one of our control parameters, with the second key control parameter being *φ*, the phase of the carrier under the envelope, which is usually abbreviated as CEP. It becomes important for few-cycle pulses, where it is well known to introduce asymmetry in the nonlinear interaction. The two typical examples are the asymmetry in the photo-electron distributions [5] and in optically induced currents [25]. In our calculations we set *σ* = 2*π*/*ω*_0_, yielding a nearly single-cycle pulse.

The system is initialized as an incoherent mixture of the two degenerate ground states:

(4) $$\rho_{0}=\frac{1}{2}\left(\left|g↑〉〈g↑|+|g↓〉〈g↓\right|\right)$$

A field directed along the *z*-axis makes the model (1) non-integrable. Each state in the mixture is propagated by integrating the time-dependent Schrödinger equation:

(5) $$\begin{array}{ll} i\frac{\partial }{\partial t}|\psi_{s}〉 & =\left({\hat{H}}_{0}+\Omega \left(t\right)\underset{i}{\sum }{\hat{\sigma }}_{i}^{z}\right)|\psi_{s}〉,\\ s & =\left\{↑,↓\right\},|\psi_{s}〉\left(0\right)=|g,s〉 \end{array}$$

where $\Omega \left(t\right)=\Omega_{0}\left(f\left(t\right)\cos\left(\omega_{0}t+\varphi \right)+\dot{f}\left(t\right)\sin\left(\omega_{0}t+\varphi \right)/\omega_{0}\right)$ , with Ω_0_ = *μ*_12_ *F*_0_ the strength of the interaction of an individual spin (or pseudospin) with the external field, *μ*_12_ the matrix element of the field interaction operator between the single-particle “up” and “down” states. In the case of a pseudospin represented by a two-level system, Ω_0_ is the peak Rabi frequency.

In practice, to integrate the Equation (5), we apply the Peierls substitution at each time step:

(6) $$\hat{U}\left(t\right)=\underset{i}{\prod }\exp\left(-iA\left(t\right){\hat{\sigma }}_{i}^{z}\right)$$

Then, the effective Hamiltonian is:

(7) $${\hat{H}}_{\text{eff}}\left(t\right)=\hat{H}\left(t\right)-i{\hat{U}}^{†}\partial_{t}\hat{U}={\hat{H}}_{0}$$

(8) $$i{\hat{U}}^{†}\partial_{t}\hat{U}=F\left(t\right)\sum {\hat{\sigma }}_{i}^{z}$$

We then apply the exponential of the static Ising Hamiltonian using a scaled Taylor expansion method [26].

(9) $$|\psi \left(t+dt\right)〉={\hat{U}}^{†}\left(t\right)\exp\left(-i{\hat{H}}_{0}dt\right)\hat{U}\left(t\right)|\psi \left(t\right)〉$$

## 3 Results

**Figure 3:**
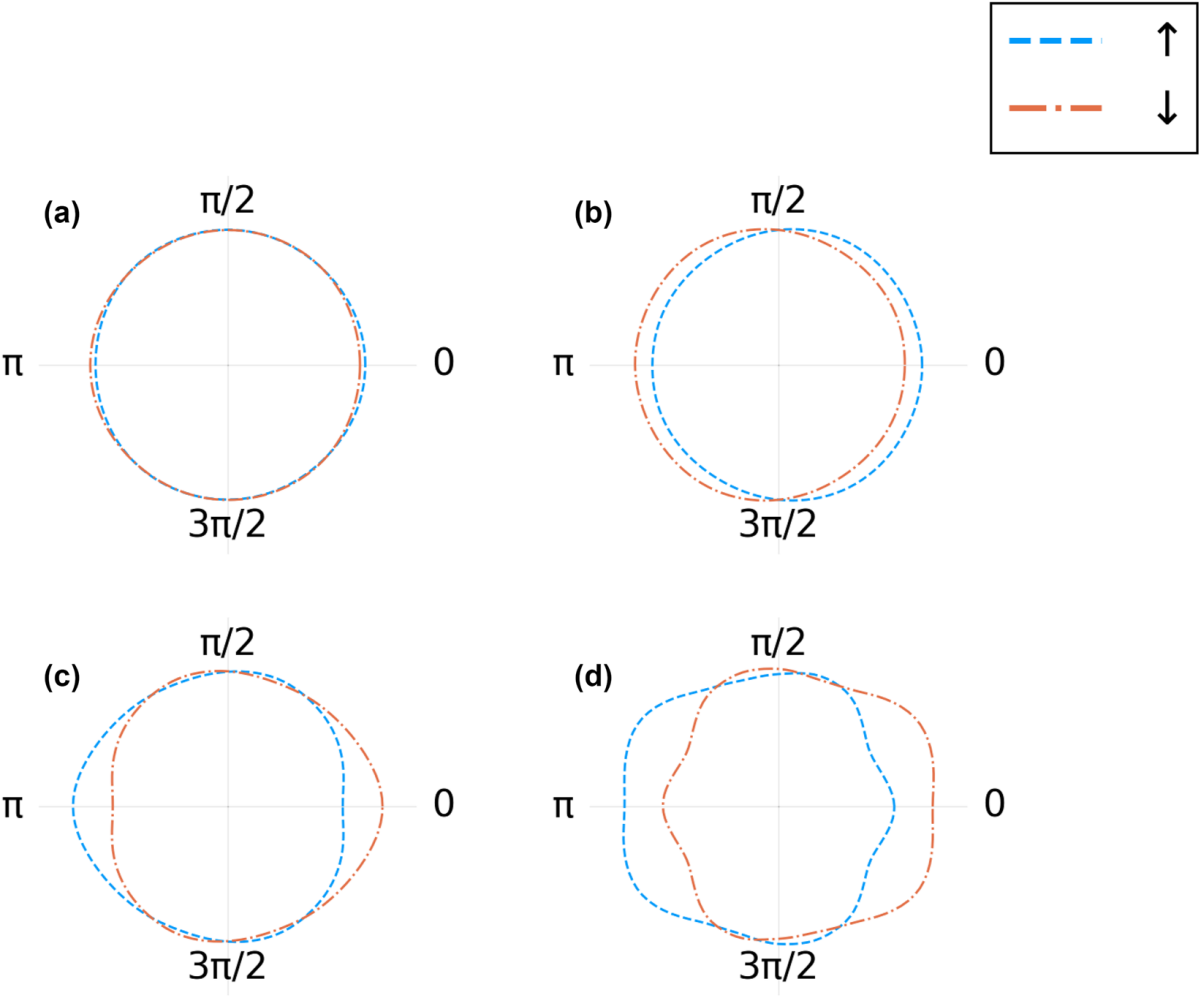
Final, CEP-dependent populations of the two ground states after excitation with the CEP controlled pulse and relaxation. The CEP is shown as a polar angle. The two curves, blue dashed and orange dot-dashed, correspond to populations in the two ground states (|*g*↑⟩, blue, and |*g*↓⟩, orange). Different panels correspond to different coupling strengths, in units of *g*: (a) Ω_0_/*g* = 0.8, (b) Ω_0_/*g* = 0.9, (c) Ω_0_/*g* = 1.0, and (d) Ω_0_/*g* = 1.2.

In the calculations, we vary the strength of the coupling Ω_0_/*g* and the phase angle *φ*. As a result, we obtain the angular dependence of the final populations of the two ground states shown in Figure 3. These results include relaxation of the excited populations to the two ground states after the end of the driving pulse. Relaxation is implemented as follows: after the system completes its unitary evolution starting from |*gs*⟩, both resulting states |*ψ*_*f*_, *s*⟩ are projected onto the eigenstates of the field-free Hamiltonian |*m*⟩: *P*_*ms*_ = ‖⟨*m*|*ψ*_*f*_, *s*⟩‖. From these states the system decays according to rate equations with the decay rates:

(10) $$W_{mn}\propto \left\{\begin{array}{cc} {\left\|\left\langle m\left|\underset{i}{\sum }\sigma_{i}^{z}\right|n\right\rangle \right\|}^{2},\; & \epsilon_{m}<\epsilon_{n}\\ 0,\; & \epsilon_{m}\geq \epsilon_{n} \end{array}\right.$$

Note that, thanks to the (*σ* ^*z*^ → −*σ* ^*z*^) symmetry inherent in the field-free Hamiltonian, its excited eigenstates also have this symmetry. While undergoing relaxation according to Equation (10), each excited eigenstate contributes to the populations of both ground states equally. Therefore, any significant asymmetry can be wholly attributed to orientation-selective excitation.

To characterize the resulting populations, we use the asymmetry value

(11) $$\begin{array}{ll} C\left(\varphi \right) & =\left(|\left\langle g↑|\psi_{f},↑\right\rangle {|}^{2}+|\left\langle g↑|\psi_{f},↓\right\rangle {|}^{2}\right)\\ & \;-\left(|\left\langle g↓|\psi_{f},↑\right\rangle {|}^{2}+|\left\langle g↓|\psi_{f},↓\right\rangle {|}^{2}\right) \end{array}$$

For sufficiently low pulse amplitudes, i.e. Ω_0_/*g* < 0.8, we observe almost no asymmetry. This situation corresponds to almost exclusively virtual excitations created in the course of the external pulse. However, once the pulse becomes sufficiently strong to create real excitations at Ω_0_/*g* ≃ 1, we observe that the final population asymmetry induced by the pulse can be as large as 0.2.

To understand the physical origin of this result, we note that the driving field frequency is small compared to the energy gap between the ground and the excited states of the system. Therefore, to interpret the dynamics, we can use the adiabatic states of the field-coupled system where the coupling Ω(*t*) is treated as slowly varying.

**Figure 4:**
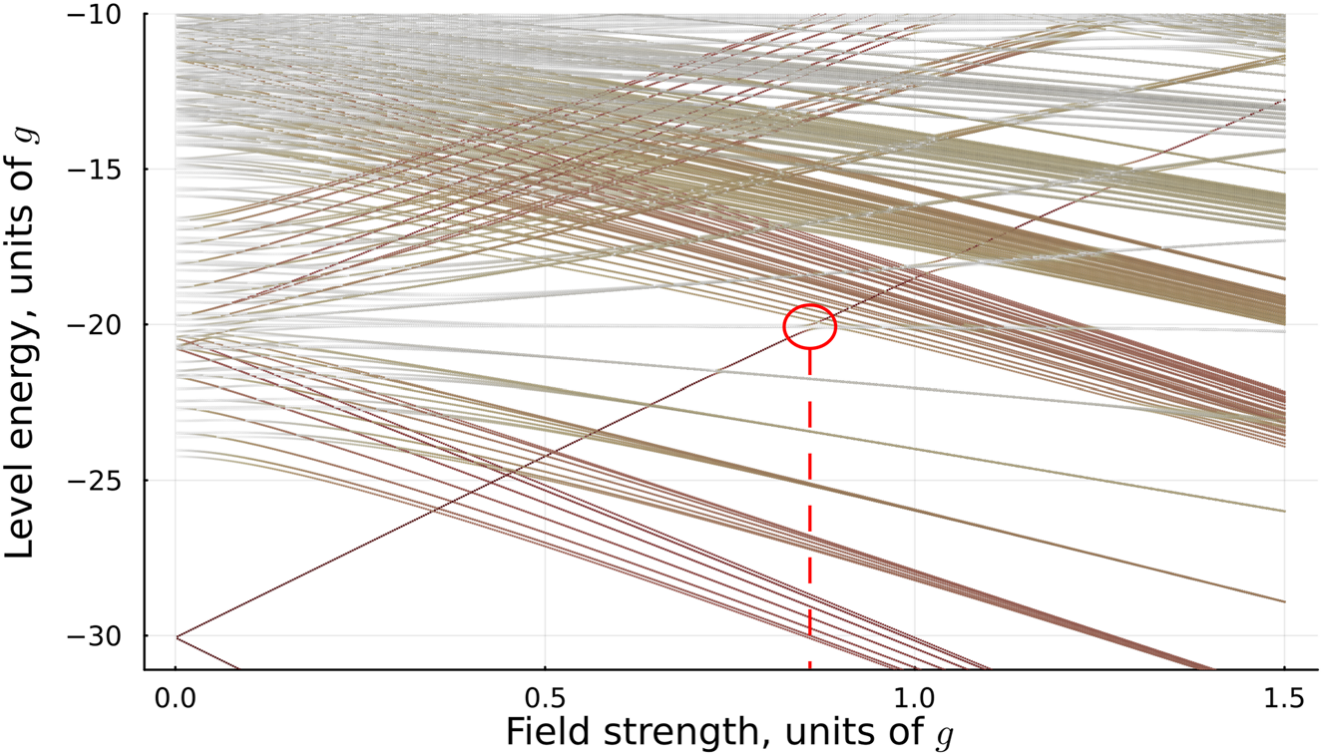
Adiabatic modifications of the states as a function of the quasistatic field coupling strength Ω, in units of *g*. During each half-cycle, the field couples the system’s excited energy states to one of the two ground states, depending on the sign of Ω, driving the depopulation of this ground state. The color saturation denotes the absolute magnitude of the average dipole value in each given state. The critical value of the coupling strength Ω ≃ 0.8 is marked with a vertical line and the corresponding region of the first avoided crossing is marked with a circle.

When a quasistatic field Ω(*t*) is applied, it raises the energy of the ground state that is antiparallel with respect to the field, while decreasing the energy of the other ground state as seen in Figure 4. As the state is driven through avoided crossings, the field couples the ground state to different excited states. The system undergoes multiple Landau–Zener–Dykhne type transitions which depopulate the affected ground state. In the opposite half-cycle, the situation is reversed. Adjacent half-cycles of the pulse drive different ground states, with the slight asymmetry between the peak values of the field giving rise to the CEP-dependent orientation-selective excitation.

**Figure 5:**
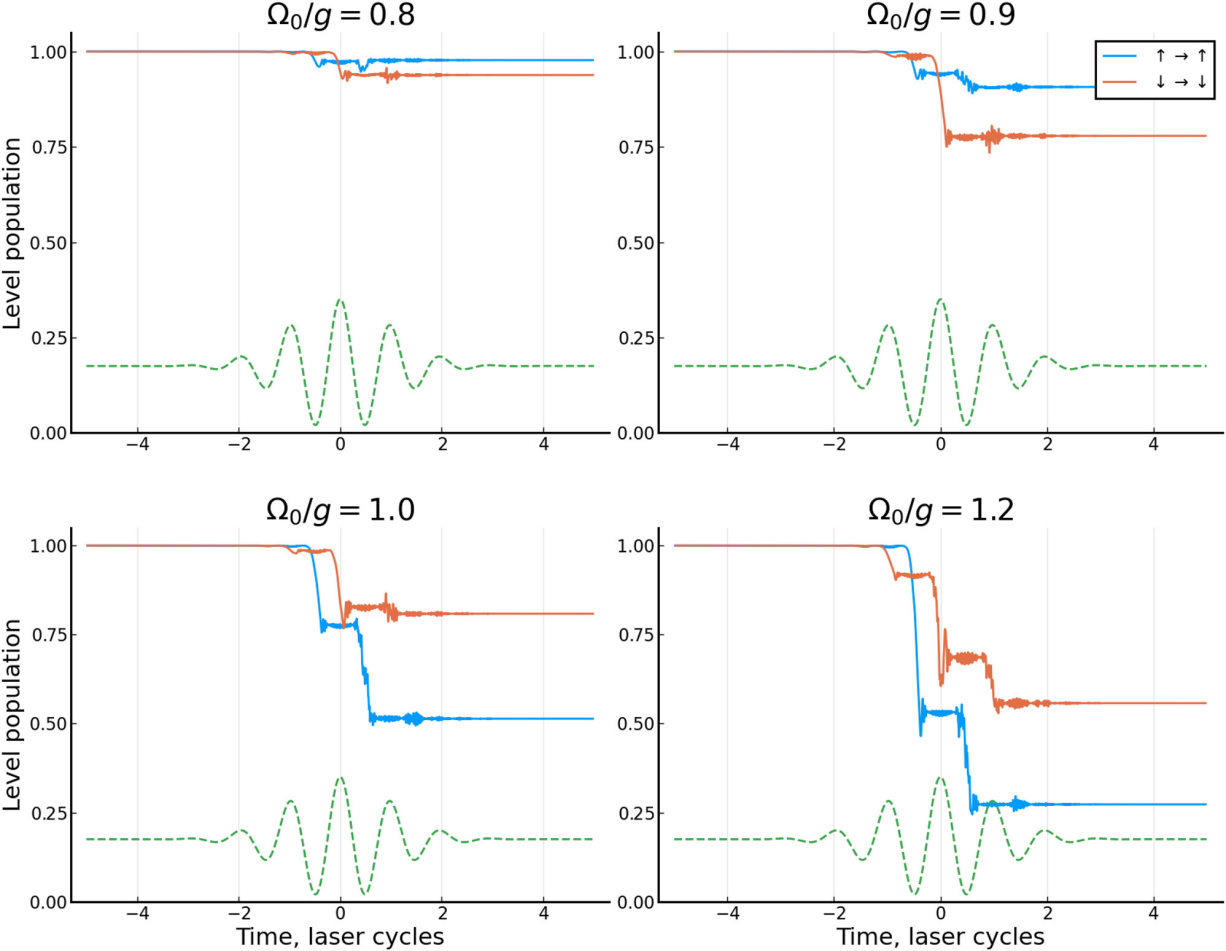
Time-dependent depopulation dynamics of the two ground states after averaging over the equal mixture of the two initial ground states, for different peak coupling strengths Ω_0_/*g*. The green curve shows the corresponding coupling field shape Ω(*t*). The CEP is set to *φ* = 0 in all cases.

The above mechanism demonstrates a frequency-domain representation of the ensuing critical behavior. Its time-domain counterpart is illustrated in Figure 5. We observe that for Ω_0_/*g* = 0.8, 1.0, the system shows fairly straightforward excitation dynamics with monotonous dependence on the field amplitude. Note that the driving field (and the nonlinear interaction with it) is perfectly symmetric for *φ* = *π*/2 and 3*π*/2, thus no asymmetry is observed for these CEP values. The situation changes above Ω_0_/*g* ≃ 1.0, when we observe rapid oscillatory dynamics emerging around the avoided crossings. The corresponding Landau–Zener–Dykhne transitions therefore act as an ultrafast clock, mapping the speed with which the system is driven through the avoided crossings onto the final populations. The real excitations created during such transitions interfere with those created during the subsequent half-cycles. The symmetry between the subsequent half-cycles is further broken by significant excitation probability during one half-cycle. These effects give rise to the population asymmetry observed at CEP *φ* = *π*/2, 3*π*/2 for Ω_0_/*g* = 1.2. In other words, for sufficiently large values of Ω_0_/*g* above the critical field, the total excitation amplitude results from interference of multiple commensurate excitation bursts, each depleting the ground state amplitude and thus breaking the time-reversal symmetry [27] inherent in the incident Gaussian pulse with CEP *π*/2, 3*π*/2. The system is now able to distinguish whether the “up” or the “down” field peak arrives first.

Importantly, the efficiency of the method grows exponentially with each consecutive CEP-controlled pulse, while the tunable interaction strength allows for a flexible trade-off between the total energy that is imparted onto the system and the number of excitation–relaxation cycles necessary to achieve conversion into a desired ground state with the desired fidelity.

## 4 Conclusion

In a recent work [28], ultrafast manipulation of spin currents in antiferromagnetic materials has been demonstrated experimentally. Another work [29] shows that spin currents excited in an antiferromagnetic layer exert torque on ferromagnetic spins when coupled through an AFM/FM interface, therefore rotating the exchange bias of the bilayer. We demonstrate that CEP-stable pulses can also be used to manipulate ferromagnetic materials directly. Therefore, the combination of our result and those of Ref. [28] shows that tailored coherent superpositions of orthogonally polarized CEP-controlled pulses can thus be used to set the direction of the exchange bias by exciting both parts of an AFM/FM interface in a controllable and ultrafast way.

Our effect can be observed using terahertz magnetic pulses [21] for such physical realizations of Ising chains as, e.g., cold atom lattices or solid-state systems described by the Hubbard model [16]. An experimental demonstration will potentially pave the way to a robust memory bit that can be both switched and read out in an all-optical way.
